# A Potent Antifungal Agent for Basal Stem Rot Disease Treatment in Oil Palms Based on Chitosan-Dazomet Nanoparticles

**DOI:** 10.3390/ijms20092247

**Published:** 2019-05-07

**Authors:** Farhatun Najat Maluin, Mohd Zobir Hussein, Nor Azah Yusof, Sharida Fakurazi, Abu Seman Idris, Nur Hailini Zainol Hilmi, Leona Daniela Jeffery Daim

**Affiliations:** 1 Institute of Advanced Technology, Universiti Putra Malaysia, 43400 UPM, Serdang, Selangor, Malaysia; farhatunnajat@yahoo.com (F.N.M.); azahy@upm.edu.my (N.A.Y.); 2 Department of Chemistry, Faculty of Science, Universiti Putra Malaysia, 43400 UPM, Serdang, Selangor, Malaysia; 3 Department of Human Anatomy, Faculty of Medicine and Health Sciences, Universiti Putra Malaysia, 43400 UPM, Serdang, Selangor, Malaysia; sharida@upm.edu.my; 4 Malaysian Palm Oil Board (MPOB), 6, Persiaran Institusi, Bandar Baru Bangi, 43000, Kajang, Selangor, Malaysia; idris@mpob.gov.my (A.S.I.); hailini@mpob.gov.my (N.H.Z.H.); 5 Sime Darby Technology Centre Sdn. Bhd., UPM-MTDC Technology Centre III, Lebuh Silikon, Universiti Putra Malaysia, 1st Floor, Block B, 43400, Serdang, Selangor, Malaysia; leona.daniela.jefferydaim@simedarbyplantation.com

**Keywords:** agronanoparticles, fumigant, anti-fungal, *Ganoderma boninense*, basal stem rot

## Abstract

The use of nanotechnology could play a significant role in the agriculture sector, especially in the preparation of new-generation agronanochemicals. Currently, the economically important plant of Malaysia, the oil palm, faces the threat of a devastating disease which is particularly caused by a pathogenic fungus, *Ganoderma boninense.* For the development of an effective antifungal agent, a series of chitosan nanoparticles loaded with a fumigant, dazomet, were prepared using various concentrations of sodium tripolyphosphate (TPP)—2.5, 5, 10, and 20 mg/mL, abbreviated as CDEN2.5, CDEN5, CDEN10, and CDEN20, respectively. The effect of TPP as a crosslinking agent on the resulting particle size of the synthesized nanoparticles was investigated using a particle size analyzer and high-resolution transmission electron microscopy (HRTEM). Both methods confirmed that increasing the TPP concentration resulted in smaller particles. In addition, in vitro fumigant release at pH 5.5 showed that the release of the fumigant from the nanoparticles was of a sustained manner, with a prolonged release time up to 24 h. Furthermore, the relationship between the chitosan-dazomet nanoparticles and the in vitro antifungal activity against *G. boninense* was also explored, where the nanoparticles of the smallest size, CDEN20, gave the highest antifungal efficacy with the lowest half maximum effective concentration (EC_50_) value of 13.7 ± 1.76 ppb. This indicates that the smaller-sized agronanoparticles were more effective as an antifungal agent. The size can be altered, which plays a crucial role in combatting the *Ganoderma* disease. The agronanoparticles have controlled release properties and high antifungal efficacy on *G. boninense*, thus making them a promising candidate to be applied in the field for *Ganoderma* treatment.

## 1. Introduction

Nanotechnology can be defined as the designation, characterization, production, and application of systems and devices by controlling their shape and size at the nanometer scale [1]. As a result, nanotechnology provides promising development in the agriculture sector. Over the past decade, researchers have been concerned with and searching for solutions to several agricultural challenges, including sustainability, crop protection, increasing productivity, and disease management [2,3,4,5]. One of the aims of nanotechnology in agriculture is to reduce the agrochemicals used by the efficient delivery of active ingredients, increasing the yield, and minimizing nutrients lost during fertilization [6]. 

The *Ganoderma* disease is one of the most serious threats to oil palm plantations, particularly in Southeast Asian countries. The fungus, *Ganoderma boninense*, is detected only when the plant is 50% internally infected by the fruiting body [7]. Once the plant is infected, the disease will significantly reduce the oil palm yield and eventually kill the plant. With the oil palm as an important commodity crop, Malaysia suffers millions in losses every year due to this disease. Currently, this disease is controlled by the removal of infected palms [3], soil mounding [4], biological control agents such as *Trichoderma* spp. [8,9] and endophyte bacteria [10], as well as systemic chemical fumigant treatment [11]. However, to date, no single treatment or control has proven to be able to effectively curb this disease in the field. 

Previous studies have shown that the fumigant treatment of dazomet was effective against *G. boninense*. Dazomet is a microgranular fumigant which releases methyl isothiocyanate (MITC) when it comes into contact with water. This MITC has been reportedly used in a broad spectrum of activities, such as inhibiting the activity of pathogenic fungi [12]. It was claimed that the MITC released by dazomet could inhibit the growth of *G. boninense* in in vitro antifungal activity studies as well as in infected palms [13]. 

Chitosan (CS) is the second most abundant polysaccharide, which can be found in a variety of different organisms, such as crabs, lobsters, and shrimp. Chitosan has been proven to have antifungal activity against *Aspergillus niger* [14], *Alternaria alternata* [15], *Rhizopus oryzae* [16], *Phomopsis asparagi* [17], and *Rhizopus stolonifer* [18]. Chitosan offers advantages such as nontoxic, biodegradable, biocompatible, and antimicrobial and antioxidant activity [19]. Chitosan nanoparticles can easily be prepared by using the ionic gelation method using tripolyphosphate (TPP) as a crosslinking agent [20,21]. The advantage of this method is attributed to its nontoxic and multivalent anion. TPP can interact with cationic chitosan by electronic forces. It was also reported that TPP could be used to control nanoparticle size and drug loading [21,22].

Nanotechnology enables a fumigant active agent such as dazomet to be encapsulated into the chitosan matrix to form a fumigant nanocarrier system and transport it to the target cells (*Ganoderma* fungus) more effectively. Thus, this study aimed to investigate the optimized parameters in synthesizing chitosan-dazomet nanoparticles using various concentrations of sodium TPP to control the particle size distribution, loading content, and encapsulation efficiency of dazomet, and subsequently, to use the nanoparticles for controlling the *Ganoderma* disease in oil palm. In addition, the effect of the size of chitosan-dazomet nanoparticles on their in vitro antifungal activity against *G. boninense*, together with the release behavior of dazomet, was also the focus of this study.

## 2. Results and Discussions

### 2.1. Nanoparticle Characterizations

#### 2.1.1. Reaction Yield and Dazomet Loading Encapsulation Efficiency

As listed in Table 1, the optimum reaction yield was observed in 5 mg/mL of TPP, where the yield remained statistically similar even after increasing the concentration of TPP. The lowest reaction yield was obtained at the lowest concentration of 2.5 mg/mL. Apart from that, the results on the loading content (LC) and encapsulation efficiency (EE) of dazomet revealed that there was no specific trend observed when the TPP concentrations were increased. The LC and EE reached a maximum at 5 mg/mL but decreased at higher concentrations of 10 and 20 mg/mL TPP. This might be due to the smaller nanoparticle sizes of dazomet-loaded chitosan nanoparticles with a 10-mg/mL concentration of sodium TPP (CDEN10) and CDEN20 (the sizes are discussed later). As reported in the previous study, a smaller particle size resulted in a lower percentage loading [23,24].

#### 2.1.2. X-ray Diffraction

**Figure 1:**
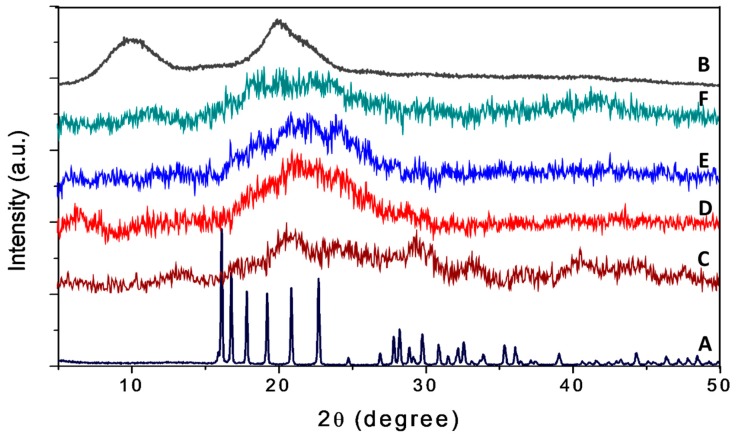
Powder XRD patterns of (**A**) pure dazomet, (**B**) chitosan, and chitosan-dazomet nanoparticles prepared at various concentrations of tripolyphosphate (TPP): (**C**) 2.5, (**D**) 5, (**E**) 10, and (**F**) 20 mg/mL.

As shown in Figure 1, pure dazomet showed a sharp peak, suggesting it is highly crystalline in nature. In contrast, chitosan showed a broad peak, showing it is an amorphous type of material. For the nanoparticles CDEN2.5, CDEN5, CDEN10, and CDEN20, a broad amorphous peak was observed, suggesting the high content of the chitosan phase, in which the crystalline peak of dazomet was buried underneath when they were encapsulated within the chitosan nanoparticles. The broad peaks at diffraction angles (2θ) of 16.1°, 16.8°, 17.8°, 19.2°, 20.8°, 24.7°, 29.8°, 30.9°, 32.1°, 32.5°, 35.3°, and 39.0° matched with the peak pattern of pure dazomet, thus suggesting that the encapsulation of dazomet into the chitosan matrix had occurred. 

#### 2.1.3. FTIR Spectroscopy

**Figure 2:**
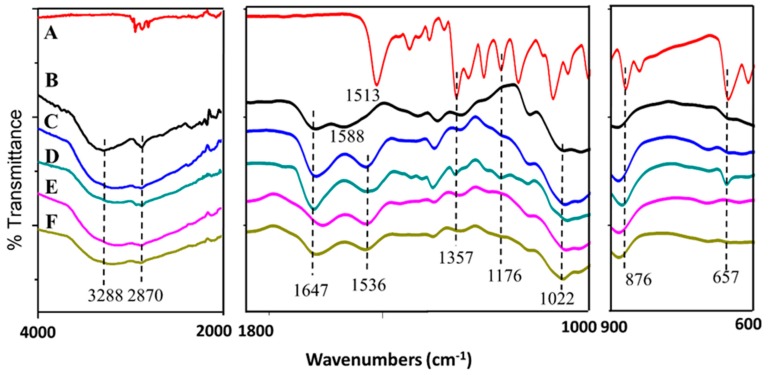
FTIR spectrum of (**A**) pure dazomet, (**B**) chitosan, and chitosan-dazomet nanoparticles prepared at various concentrations of TPP: (**C**) 2.5, (**D**) 5, (**E**) 10, and (**F**) 20 mg/mL.

As shown in Figure 2, broad bands at 3288 and 2870 cm^−1^ were due to the NH_2_ stretching and C–H bond of the chitosan, respectively [25]. Chitosan also showed characteristic broad bands at 1647, 1588, and 1022 cm^−1^, which indicated the stretching of the CO–NH_2_ group, NH_2_ bending, and C–O–C stretching vibration, respectively [25]. For the synthesized nanoparticles CDEN2.5, CDEN5, CDEN10, and CDEN20, all showed the characteristic bands of chitosan with the slight shifting of NH_2_ at 1536 cm^−1^. The shifting might be due to *n*–H bending of dazomet at 1513 cm^−1^ [26]. Additional bands of dazomet can be seen for the synthesized nanoparticles (Figure 2C–F) at 1357, 1176, 876, and 657 cm^−1^, which can be attributed to the C–*n* stretching, C=S stretching, and C–H bending of 1,3,5 trisubstituted aromatic alkane, respectively, thus suggesting the encapsulation of dazomet into the chitosan matrix [26,27].

#### 2.1.4. Thermal Analysis

**Figure 3:**
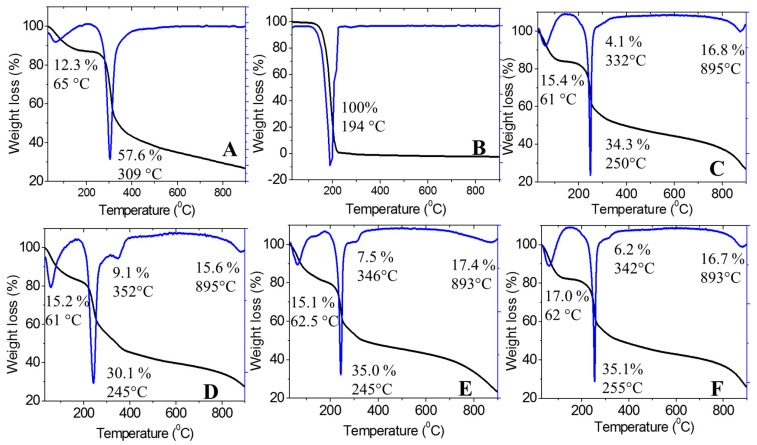
Thermogravimetric (black curve) and differential thermogravimetric (blue curve) (TGA/DTG) thermograms of (**A**) chitosan, (**B**) pure dazomet, and chitosan-dazomet nanoparticles prepared at various concentrations of TPP: (**C**) 2.5, (**D**) 5, (**E**) 10, and (**F**) 20 mg/mL.

The thermal stability of the synthesized nanoparticles was studied using a thermal analyzer, and the thermogravimetric and differential thermogravimetric (TGA/DTG) thermograms are shown in Figure 3. This analysis provided quantitative information about the components in the synthesized chitosan-dazomet nanoparticles. Chitosan showed two stages of weight loss at 65 and 309 °C, which were attributed to the release of water molecules and decomposition of chitosan (loss of hydrogen bonding), respectively. In addition, at the end of the analysis, nearly 30% of the sample remained as a residue, indicating the higher thermal stability of chitosan. One hundred percent weight loss was obtained at 194 °C for pure dazomet, which indicated the total decomposition of dazomet. 

The nanoparticles of CDEN2.5, CDEN5, CDEN10, and CDEN20 showed a similar pattern with four stages of weight loss. For the first stage at around 60 °C, weight loss occurred due to the release of water molecules. The second stage at 245–255 °C was attributed to the decomposition of chitosan. The third stage, at 332–352 °C, was due to the decomposition of dazomet, thus showing the higher thermal stability of dazomet in the CDEN2.5, CDEN5, CDEN10, and CDEN20 nanoparticles compared with their pure dazomet. For the last stage at around 890 °C, the weight loss was attributed to the char due to the decomposition of chitosan. 

#### 2.1.5. Morphology and Particle Size Distribution

**Figure 4:**
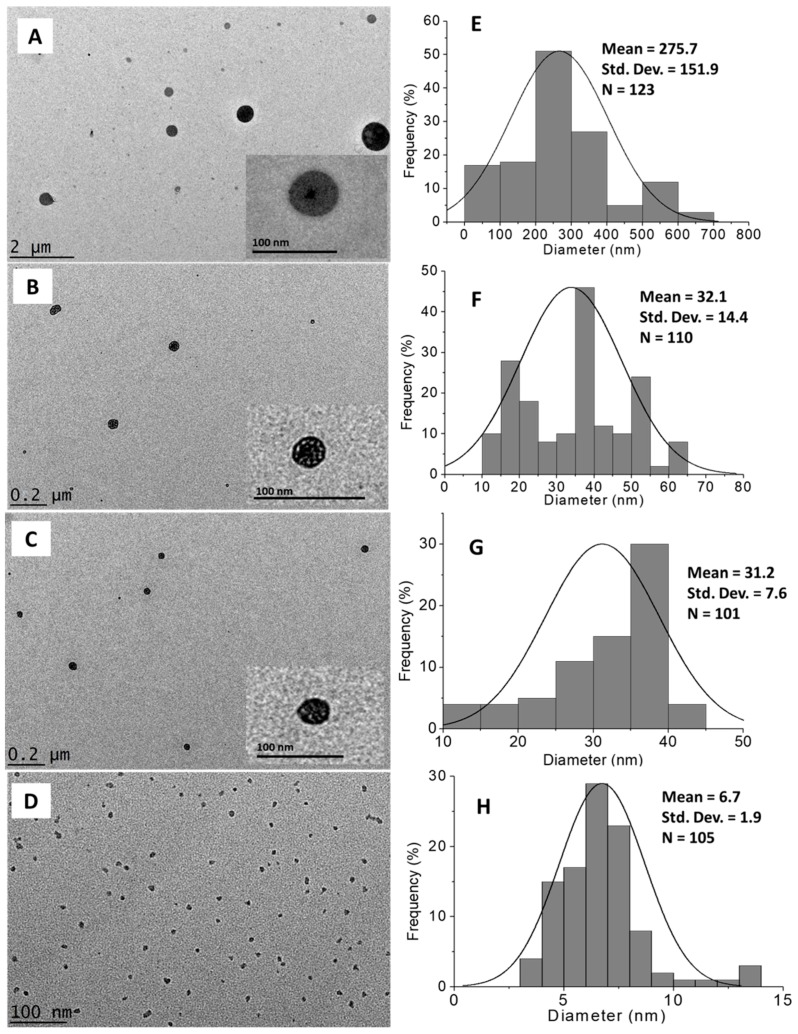
High-resolution transmission electron microscopy (HRTEM) images of the synthesized dazomet-loaded chitosan nanoparticles (CDENs) prepared at various concentrations of TPP: (**A**) 2.5, (**B**) 5, (**C**) 10, and (**D**) 20 mg/mL and their particle size distributions: (**E**) 2.5, (**F**) 5, (**G**) 10, and (**H**) 20 mg/mL.

The morphological and particle size distribution of CDEN2.5, CDEN5, CDEN10, and CDEN20 were studied by high-resolution transmission electron microscopy (HRTEM) (Figure 4A–D), and their size distribution was measured via ImageJ software (Figure 4E–H). As shown in the Figure 4, a sphere shape was obtained for all the synthesized nanoparticles. In addition, the effect of the concentration of TPP could be observed, where the mean diameter size became smaller as the concentration of TPP was increased. At the lowest concentration of 2.5 mg/mL, CDEN2.5 showed the relatively largest sphere particle with a mean diameter of 275.7 nm, followed by CDEN5 and CDEN10 (5 and 10 mg/mL TPP) with a mean diameter of 32.1 and 31.2 nm, respectively. Moreover, at the highest TPP concentration of 20 mg/mL, CDEN20 showed the relatively smallest sphere particle size with a mean diameter of 6.7 nm.

**Figure 5:**
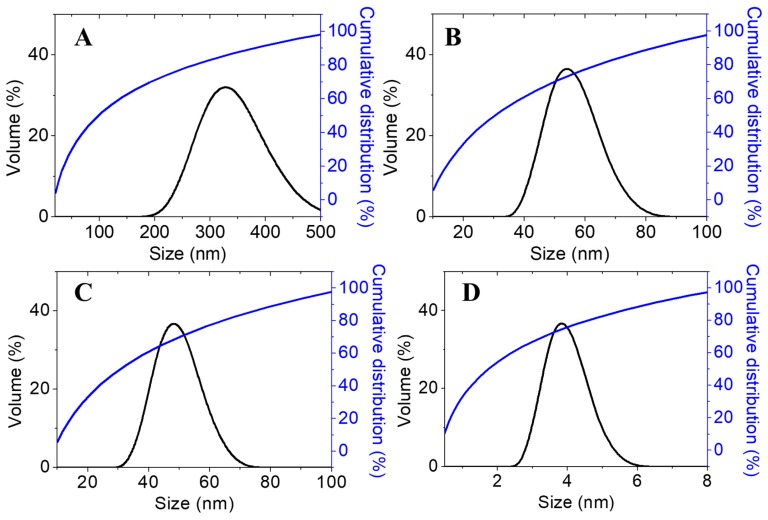
Cumulative and relative particles size distributions (PSDs) of chitosan-dazomet nanoparticles prepared at different concentrations of TPP: (**A**) 2.5, (**B**) 5, (**C**) 10, and (**D**) 20 mg/mL.

Furthermore, the particle size distribution in the solvated state was measured, in which solvent molecules (deionized water) interacted with the particles. As shown in Figure 5, the same trend can be observed, where increasing the TPP concentration resulted in the smaller size of the synthesized nanoparticles, presumably due to the adsorption of oppositely charged ions in the solvent medium (deionized water). It is known that the formation of CS-TPP nanoparticles is based on the interaction between free amino groups in chitosan, where –NH_2_ is protonated to –NH_3_^+^ under the acid condition with the negative charge of the multivalent anion, TPP. Thus, when the TPP concentration was increased, more inter- and intramolecular crosslinking happened between chitosan and TPP, thus resulting in smaller particles sizes [21]. 

### 2.2. In Vitro Dazomet Release

**Figure 6:**
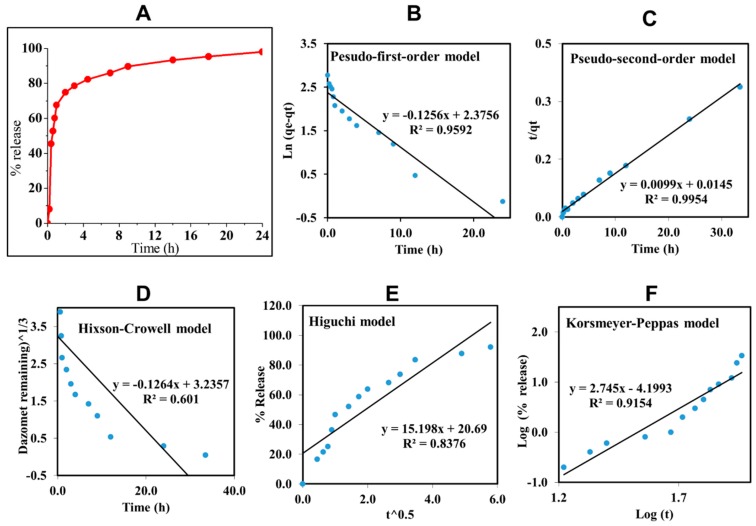
Cumulative release profiles of dazomet from CDEN5 nanoparticles at pH 5.5 (**A**) and their fitting of the data using five different mathematical models at pH 5.5 (**B**–**F**).

To study the delivery behavior of dazomet in response to time, CDEN5 was incubated in a phosphate buffer saline solution at pH 5.5. CDEN5 was chosen for this study due to its highest loading of dazomet compared with the others. As shown in Figure 6, CDEN5 showed a burst effect in the first 4 h, maybe due to the dazomet which was adsorbed close to the surface of the sphere of its nanoparticles. Thereafter, the sustained release of dazomet was achieved up to 24 h with a 97.8% cumulative release.

In order to design a more effective nanodelivery system, it is important to determine the active ingredient release profiles using kinetic models such as the pseudo-first-order and pseudo-second-order kinetics and other mathematical models such as Higuchi, Hixson–Crowell, and Korsmeyer–Peppas models. By fitting the data of the dazomet release from the nanoparticles into the five different kinetic and mathematical models, the linear fits of the models were obtained, as presented in Figure 6B–F.

The linear form in the first-order kinetic model is given in Equation (1), where q_e_ and q_t_ are the quantities of dazomet released at equilibrium and at any time (t), respectively, and k_1_ is the rate constant for the pseudo-first-order release kinetics. The linear form in the second-order kinetic model can be represented by Equation (2), where K_2_ is the rate constant of the pseudo-second-order release kinetics. The Higuchi model (Equation (3)) describes the increased release of the dazomet from the nanoparticles with an increasing square root of time, where K_H_ is the Higuchi rate constant. The Hixson–Crowell model (Equation (4)) provides a relationship between the cube root of the remaining dazomet left in the nanoparticles as a function of time, where K_HC_ is the Hixson–Crowell rate constant, M_o_ is the initial quantity of the dazomet in the nanoparticles, and q_t_ is the quantity released at time t. The Korsmeyer–Peppas (Equation (5)) model provides a relationship between the log of the percentage of the dazomet released and the log of time, where q_∞_ is the release at the infinite time and *n* is the release exponent.

Ln (q_e_ − q_t_) = ln q_e_ − K_1_t (1)

t/q_t_ = 1/K_2_q^2^_e_ + t/q_e_ (2)

q_t_ = K_H_ √t (3)

^a^√M_0_ − ^a^√q_t_ = K_HC_t (4)

qt/q∞ = Kt^n^ (5)

The calculated correlation coefficient (*R*^2^) of the release data revealed that the release kinetics of CDEN5 fit well to the pseudo-second-order kinetics (*R*^2^ = 0.9954) compared with the other models used in this work. This indicated that the overall reaction was dependent upon the ion exchange between the dazomet molecules and the release medium at the time of release and at the equilibrium with the rate constant (K_2_) of 0.0099 mg h^−1^ and t_1/2_ of 11.16 h [28,29].

### 2.3. Antifungal Efficacy on G. boninense

#### 2.3.1. In Vitro Antifungal Activity Assay 

The antifungal efficacy for the inhibition of *G. boninense* was evaluated by incubating potato dextrose agar (PDA) with only the solvent (control), chitosan, pure dazomet, and the synthesized nanoparticles (CDEN2.5, CDEN5, CDEN10, and CDEN20). Their inhibitory effect was then evaluated based on the inhibition rate and the calculated half maximum effective concentration (EC_50_) value, where the higher the inhibition rate, the better the antifungal activity against *G. boninense*, or the lower the EC_50_ value, the more effective the fumigant at killing *G. boninense*.

**Figure 7:**
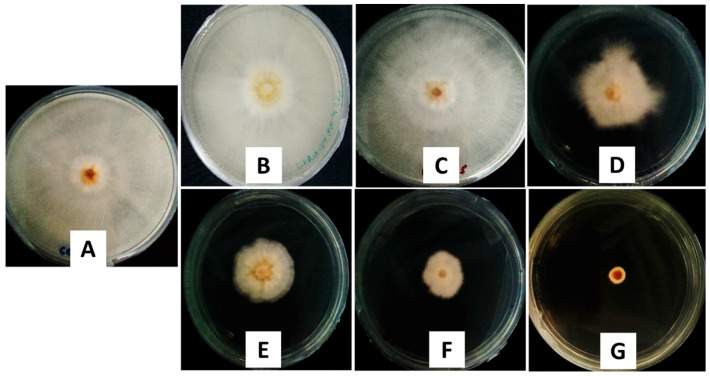
Antifungal effect on *G. boninense* of the (**A**) control, (**B**) chitosan, (**C**) pure dazomet, and chitosan-dazomet nanoparticles prepared at various concentrations of TPP: (**D**) 2.5, (**E**) 5, (**F**) 10, and (**G**) 20 mg/mL at 50 ppb concentration, 7 days after incubation at 28 ± 2 °C.

The antifungal activity was analyzed using the mycelia growth method. As shown in Figure 7, on day 7, at a concentration of 50 ppb, the control and chitosan showed no inhibitory effect as the maximum mycelial growth was achieved (radius of 40.00 mm), while pure dazomet showed a low inhibitory effect with a radius of 30.88 mm. On the other hand, remarkable inhibitory effects could be seen with the synthesized nanoparticles, as the mycelial growth was much lower compared with the others. Interestingly, as the concentration of TPP was increased, the size of the resulting nanoparticles became smaller, resulting in a smaller radius of mycelial mean growth: 13.25, 10.38, 5.38, and 0.63 mm for CDEN2.5, CDEN5, CDEN10, and CDEN20, respectively.

**Figure 8:**
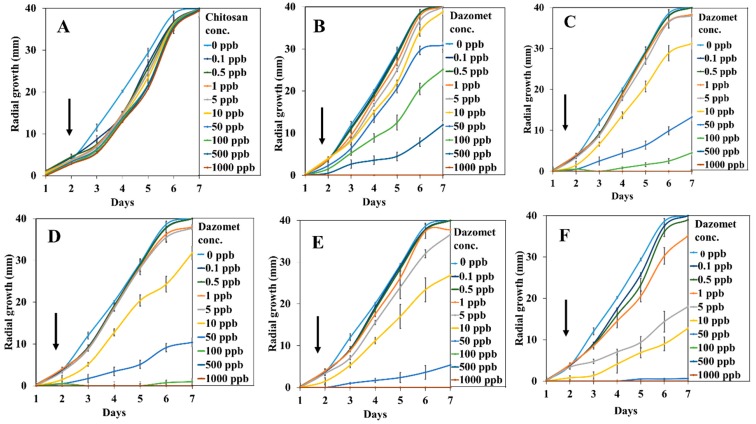
Growth curve of *G. boninense* from day 1 to 7 incubated in (**A**) chitosan, (**B**) pure dazomet, and chitosan-dazomet nanoparticles at various concentrations of TPP: (**C**) 2.5, (**D**) 5, (**E**) 10, and (**F**) 20 mg/L incubation at 28 ± 2 °C. Black arrows represent the increasing of the dazomet concentration.

**Figure 9:**
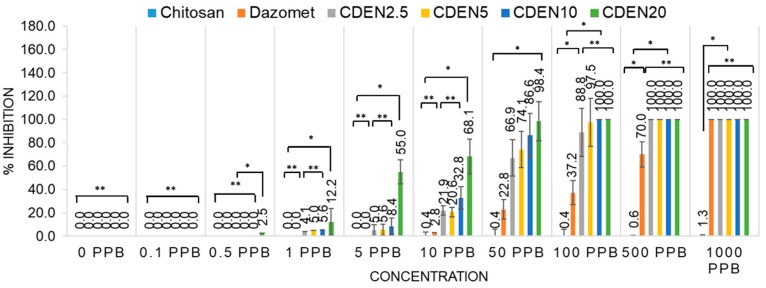
Percentage inhibition of radial growth on *G. boninense* against concentration at day 7 of incubation at 28 ± 2 °C of chitosan, pure dazomet, and chitosan-dazomet nanoparticles at various concentrations of TPP, where * *p* < 0.01 (significant) and ** *p* > 0.5 (not significant); the error bars represent standard deviation of the mean.

Figure 8 and Figure 9 show the mycelial mean radial growth curve of *G. boninense* from day 1 up to day 7 and the calculated percentage inhibition of mycelial mean radial growth (PIRG) at day 7, respectively. The minimal inhibitory effect of chitosan was observed, as the mycelial growth was almost similar to the control, whereas pure dazomet had significant inhibitory effects starting from 10 ppb (2.8% PIRG) and 100% inhibition at 1000 ppb. Moreover, enhanced inhibitory effects could be seen clearly in the synthesized nanoparticles. CDEN2.5, CDEN5, and CDEN10 showed a significant effect starting from 1 ppb with PIRG of 4.1%, 5.0%, and 5.6%, respectively. In addition, CDEN2.5 and CDEN5 achieved complete inhibition (100%) at 500 ppb, while CDEN10 at 100 ppb. Furthermore, the remarkable inhibitory effect of CDEN20 could be seen as early as at 0.5 ppb (2.5% PIRG), with complete inhibition achieved at 100 ppb. 

In addition, the EC_50_ of fumigants was determined using the Sigma Plot 10.0 software, as presented in Table 2. Chitosan showed the highest EC_50_ with a value of 1534.5 ppb, followed by pure dazomet showing the highest EC_50_ with a value of 152.2 ppb. Moreover, the synthesized nanoparticles showed better antifungal activity on *G. boninense*, where EC_50_ values of 25.4, 20.7, and 14.5 ppb were observed for CDEN2.5, CDEN5, and CDEN10, respectively. The lowest EC_50_ value with the highest antifungal activity was observed for CDEN20 with a value of only 4.6 ppb. 

**Figure 10:**
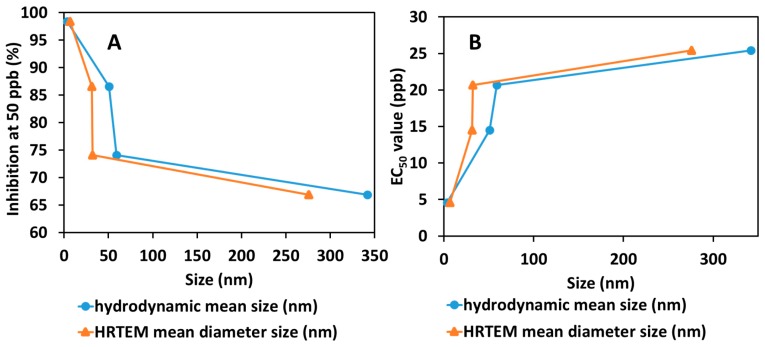
The relationship between the hydrodynamic mean particle size distribution and HRTEM mean particle size distribution of the synthesized chitosan-dazomet nanoparticles to (**A**) their percentage inhibition at 50 ppb and (**B**) the calculated EC_50_ (ppb) value on *G. boninense*.

In order to study the relationship between the size of the synthesized nanoparticles and EC_50_ value, as well as the percentage of inhibition of mean mycelial growth of *G. boninense*, a plot of the relationship was made, as shown in Figure 10. As discussed earlier, the increase in TPP concentration resulted in a decrease in the particle size. As presented in Figure 10, both methods showed that a smaller particle size resulted in a lower EC_50_ value and higher inhibition percentage. This shows that smaller particle sizes of the synthesized chitosan-dazomet nanoparticles have better antifungal activity against *G. boninense.* The results parallel previous studies which reported that smaller nanoparticles of chitosan gave higher antimicrobial activity [30,31,32]. The smaller nanoparticles imply that they have a larger surface area that can be contacted with the fungus cell, thus increasing their antifungal properties [33]. 

#### 2.3.2. Mathematical Modeling of Fungal Growth 

**Figure 11:**
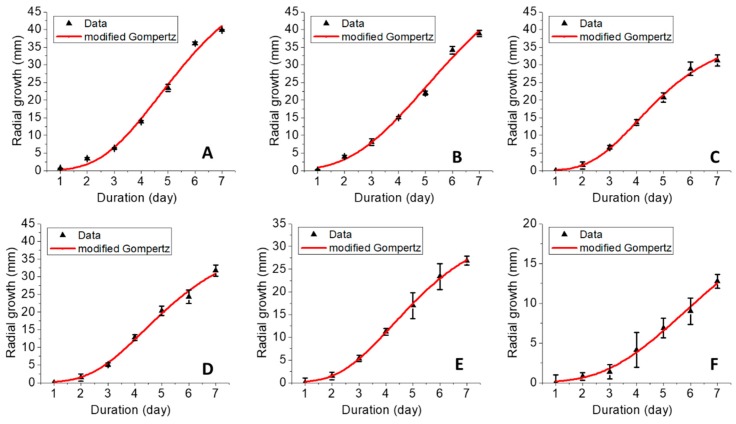
Growth curves fitted by the modified Gompertz model of *G. boninense* incubated at 10 ppb of (**A**) chitosan, (**B**) pure dazomet, and chitosan-dazomet nanoparticles at various concentrations of TPP: (**C**) 2.5, (**D**) 5, (**E**) 10, and (**F**) 20 mg/L (error bars represent standard deviation of the mean).

Analysis of the sigmoidal growth curve was performed using the modified Gompertz model (Equation (6)) as was described earlier by Halmi et al. [34]:

Y = Aexp [−exp [(μ_max_e/A) (λ − t) + 1] (6)

where A is the maximum fungal growth achieved at the stationary phase, μ_max_ is the maximum specific growth rate, e is the exponent (2.718281828), t is sampling time, and λ is lag time. The mycelial growth data at 10 ppb were chosen for this analysis and the fitting results and their data are shown in Figure 11 and Table 3, respectively. In general, *G. boninense* presented a short lag phase (<2 days), while the exponential phase lasted about 4–5 days. This study was stopped at day 7 due to the maximum mycelial growth on the petri dish being obtained for the control; thus, it was stopped before the stationary phase could be reached. The Gompertz model is a classical growth model based on the exponential relationship between specific growth rate and time [35]. Chitosan and pure dazomet presented the highest maximum fungal growth (A) and the highest maximum specific growth rate (μ_max_). Moreover, increasing the TPP concentration decreased the A and μ_max_. In addition, the lag phase (λ) achieved was statistically similar for chitosan, CDEN2.5, CDEN5, and CDEN10, while shorter λ was obtained for pure dazomet and CDEN20. 

## 3. Materials and Methods 

### 3.1. Materials

Dazomet (C_5_H_10_N_2_S_2_, molecular weight of 162.269 g/mol) was purchased from Changzhou Aiteng (Jiangsu, China) with 98% purity and was used as received. Chitosan (medium molecular weight, 190,000–310,000 degree of acetylation), TWEEN-80, and sodium TPP were purchased from Sigma Aldrich (St. Louis, MO, USA). Hydrochloric acid (37%) and *n*,*n*-dimethylformamide (DMF) were purchased from Friendemann Schmidt (Parkwood, Australia) and Merck (Kenilworth, NJ, USA), respectively. All other reagents used were of analytical grade. *G. boninense* culture was provided by the Malaysian Palm Oil Board (MPOB), Bangi, Malaysia, maintained in PDA media purchased from Oxoid, Thermo Scientific (pH 5.5) (Waltham, MA, USA), and incubated at 28 ± 2 °C.

### 3.2. Preparation of Chitosan-Dazomet Nanoparticles

CDENs were prepared using the ionic gelation method [25]. Chitosan was dissolved in a 1.0% (*v*/*v*) acetic acid solution at the concentration of 5 mg/mL. Due to its low water solubility, dazomet was dissolved in DMF (10 mg/mL) first and then added to a chitosan solution under stirring until a homogenous solution was obtained. Then, 2% *v*/*v* of TWEEN-80 was added as a surfactant. Various concentrations of sodium TPP ranging from 2.5, 5, 10, to 20 mg/mL (abbreviated as CDEN2.5, CDEN5, CDEN10, and CDEN20, respectively) were prepared in deionized water separately. The nanoparticles were formed spontaneously upon addition of 40 mL sodium TPP solution (added dropwise using a burette while stirring). The final TPP-to-chitosan ratio achieved was 1:2.5 (*v*/*v*). The resulting suspension was then centrifuged at 40,000 rpm for 10 min and then freeze-dried overnight. The chitosan-dazomet nanoparticles were obtained through the ionic gelation method based on the crosslinking of positively charged chitosan with negatively charged TPP.

### 3.3. Reaction Yield and Dazomet Loading Encapsulation Efficiency

The reaction yield of the synthesized nanoparticles was calculated using Equation 7 [36]:

RY = [Produced nanoparticle (mg)/(used chitosan (mg) + used dazomet (mg))] × 100(7)

The dazomet LC and EE were determined using a Perkin Elmer Lambda 35 UV–Vis spectrophotometer (Akron, Ohio, United States) at λ_max_ of 282 nm. Briefly, 5.0 mg of the synthesized nanoparticles were dissolved in 10.0 mL methanol and HCl (0.5% *v*/*v*) under sonication. The nanoparticles were ensured to be completely dissolved, thus releasing 100% of the dazomet content. The dazomet LC and EE were calculated according to the following equations:

LC (%) = [weight of dazomet in nanoparticles/weight of nanoparticles] × 100(8)

EE (%) = [weight of dazomet in nanoparticles/initial amount of dazomet in the system] × 100(9)

### 3.4. Dazomet Release Profile Study

The dazomet release profile from the nanoparticles was investigated using UV–Vis spectroscopy. Briefly, 30.0 mg of the synthesized nanoparticles was dispersed into 30 mL of medium and shaken in an incubator shaker (27 °C) at 100 rpm. The medium contained phosphate-buffered saline solution (PBS) at pH 5.5 (PDA media pH). At predetermined intervals, 3 mL of the solution was taken out and replaced with fresh medium. The concentration of the released dazomet was determined by a UV–Vis spectrometer at a wavelength of 282 nm.

### 3.5. Characterizations

FTIR was performed on a Thermo Nicolet Nexus spectrometer with a Smart Orbit (Waltham, MA, USA) in the range of 400–4000 cm^−1^. 

The crystallinity study was carried out by powder X-ray diffraction (PXRD), Bruker D8 Advance powder XRD (Billerica, MA, USA) using CuK_α_ radiation (λ = 0.15406 nm) at 40 kV and 40 mA.

The thermal stability and decomposition were done by TGA/DTG analysis, Mettler-Toledo 851e (Columbus, OH, USA) at a heating rate of 10 °C min^−1^ in 150-µL alumina crucibles in the range of 30–900 °C. 

The hydrodynamic particle size distribution was determined by the dynamic light scattering (DLS) method using a particle size analyzer Nano Series Nano-ZS (Malvern Panalytical Ltd., Malvern, United Kingdom). 

The internal morphology and particle size diameter were studied using HRTEM, FEI Tecnai G2 F20 S-TWIN (Hillsboro, OR, USA).

### 3.6. In Vitro Antifungal Activity Studies

The in vitro antifungal activity of the synthesized nanoparticles was evaluated against *G. boninense* using the poisoned medium technique, using PDA medium. The PDA were amended in several different conditions (pure dazomet, chitosan, CDEN2.5, CDEN5, CDEN10, and CDEN20) at several concentrations (0.1, 0.5, 1, 5, 10, 50, 100, 500, and 1000 ppb of active ingredient), which were prepared in acetone and 0.5% (*v*/*v*) HCl. Medium with the only solvent served as a control. Five millimeters of the mycelial disc from the margins of actively growing culture of *G. boninense* were placed at the center of the amended PDA. The radial growth of the mycelia was measured for 7 days of inoculation by incubating the petri plates at 28 ± 2 °C (*n* = 5). Mycelial growth was recorded every day. The PIRG of the fumigant was then calculated. The growth curve data were fitted using sigmoidal models of the modified Gompertz model using CurveExpert Professional software, version 2.6.3 using a method published elsewhere [34]. 

### 3.7. Statistical Analysis

Data are presented as mean ± standard deviation and the statistical difference of the parameters was analyzed using the ANOVA and Tukey’s test (*p* ≤ 0.05) using the SPSS software. The EC_50_ of the synthesized chitosan-dazomet nanoparticles was determined using the sigma plot analysis of Sigma Plot 10.0. 

## 4. Conclusions

In this study, various sizes of chitosan-dazomet nanoparticles were successfully synthesized by adjusting the TPP concentrations of 2.5, 5, 10, and 20 mg/mL as crosslinking agents via the ionic gelation method. Hydrodynamic mean size and HRTEM revealed that the spherical shape of CDENs decreased with the increase of TPP concentration. In addition, CDENs prepared using 5 mg/mL TPP showed the highest dazomet loading of 47.8%, followed by CDENs prepared using 10, 20, and 2.5 mg/mL, with loadings of 34.8%, 33.2%, and 28.3%, respectively. Moreover, the dazomet in CDENs prepared using 5 mg/mL TPP exhibited controlled release properties which could prolong the release time of dazomet up to 24 h. In addition, dazomet entrapped into CDENs exhibited better thermal stability compared with its counterpart, pure dazomet. Furthermore, the antifungal study against *G. boninense* with CDENs showed outstanding inhibition with lower EC_50_ values compared with the pure dazomet. CDENs prepared using 20 mg/mL TPP showed the lowest EC_50_ value, with the highest inhibitory activity against *G. boninense* at 13.7 ± 1.76 ppb. CDENs prepared using 10, 5, and 2.5 mg/mL TPP showed EC_50_ values of 32.1 ± 1.37, 34.0 ± 0.52, and 34.7 ± 0.22 ppb, respectively, thus suggesting that the smaller the particle size, the higher the antifungal activity of the resulting chitosan-fumigant nanoparticles. 

## Figures and Tables

**Table 1:** Reaction yield, loading content, and encapsulation efficiency of the synthesized nanoparticles.

Synthesized Nanoparticles	Reaction Yield * (%)	Loading Content * (%)	Encapsulation Efficiency * (%)
CDEN2.5	60.5 ± 2.5^a^	28.3 ± 3.5^a^	78.3 ± 5.0^a^
CDEN5	76.0 ± 3.0^b^	47.8 ± 2.0^b^	97.8 ± 2.5^b^
CDEN10	73.3 ± 2.0^b^	34.8 ± 4.0^c^	84.8 ± 3.0^c^
CDEN20	75.0 ± 3.5^b^	33.2 ± 2.0^c^	83.2 ± 4.5^c^

* Different letters in the same column indicate significant differences between means (*p* ≤ 0.05) according to Tukey’s test.

**Table 2:** Calculated half maximum effective concentration (EC_50_) of chitosan, pure dazomet, and chitosan-dazomet nanoparticles prepared at various concentrations of TPP on *G. boninense* at day 7 of incubation at 28 ± 2 °C.

Parameters	Type of Fumigants					
	Chitosan	Pure Dazomet	CDEN2.5	CDEN5	CDEN10	CDEN20
**EC_50_ (ppb)**	1534.5	152.2	25.4	20.7	14.5	4.6
**Fiducial limit (ppb)** **(lower–upper)**	494.0–13280.4	73.2–565.8	12.8–63.6	8.5–76.0	7.2–38.6	3.4–6.6

**Table 3:** Fitted growth parameters according to the modified Gompertz model.

Parameters	Type of Fumigants					
	Chitosan	Pure Dazomet	CDEN2.5	CDEN5	CDEN10	CDEN20
A *	57.75 ± 10.84^a^	64.79 ± 14.34^a^	38.21 ± 2.59^b^	40.53 ± 5.16^b^	34.90 ± 1.54^b^	24.65 ± 7.57^c^
μ_max_ * (day ^−1^)	45.90 ± 0.32^a^	43.51 ± 0.19^a^	32.41 ± 0.21^b^	32.33 ± 0.27^b^	27.48 ± 0.10^c^	17.86 ± 0.20^d^
λ * (day)	1.46 ± 0.11^a^	1.36 ± 0.08^b^	1.57 ± 0.07^a^	1.50 ± 0.09^a^	1.50 ± 0.03^a^	1.34 ± 0.09^b^
**R^2^**	0.992	0.994	0.997	0.995	0.999	0.994

* Different letters in the same row indicate significant differences between means (*p* ≤ 0.05) according to Tukey’s test.
